# Neuroanatomy of Anxiety: A Brief Review

**DOI:** 10.7759/cureus.2055

**Published:** 2018-01-12

**Authors:** Cameron K Schmidt, Shehzad Khalid, Marios Loukas, R. Shane Tubbs

**Affiliations:** 1 Clinical Anatomy, Seattle Science Foundation; 2 Department of Anatomical Sciences, St. George's University School of Medicine, Grenada, West Indies; 3 Neurosurgery, Seattle Science Foundation

**Keywords:** anxiety disorders, neuroanatomy, functional imaging

## Abstract

Anxiety disorders are among the most prevalent psychological issues worldwide, displaying the youngest age of onset and greatest chronicity of any mood or substance abuse disorder. Given the high social and economic cost imposed by these disorders, developing effective treatments is of the utmost importance. Anxiety disorders manifest in a variety of symptomatic phenotypes and are highly comorbid with other psychological diseases such as depression. These facts have made unraveling the complex underlying neural circuity an ever-present challenge for researchers. We offer a brief review on the neuroanatomy of anxiety disorders and discuss several currently available therapeutic options.

## Introduction and background

Anxiety is understood as an adaptive response, serving to maximize survival through the avoidance of potentially harmful events [1]. In unraveling the complexities of anxiety, it is important to distinguish anxiety from fear. Fear is a response triggered by the presence of an imminent, real threat, whereas anxiety revolves around the anticipation of potential harm in the future [2]. While anxiety is a necessary tool for human cognition, anxiety disorder describes the uncontrolled, excessive persistence of anxious responses such that an individual is no longer able to live a normal functioning life.

The lifetime prevalence of anxiety disorders is estimated to be from 11.3% to 14.7% worldwide [3]. Epidemiologic data from the World Health Organization (WHO) estimates the lifetime prevalence for anxiety disorders to be 25% in the United States [4]. As of 2010, anxiety disorders were the most common mental disorder in the European Union, estimated to have an annual cost of 74.4 billion euros [5].

The prevalence of anxiety disorders varies by culture, with rates in Euro/Anglo cultures almost double what they are in African cultures [6]. Prevalence rates vary too by gender, with women having a statistically greater likelihood than men of developing an anxiety disorder at some point in their lives [3]. Finally, and perhaps most importantly, anxiety disorders display the youngest age of onset and greatest chronicity of any mood or substance abuse disorders [4].

Given the early age of onset, chronicity, and high rate of comorbidity associated with anxiety disorders, early interventions may prevent the development of many secondary disorders. With the high social and economic cost imposed by anxiety disorders, the development of effective treatments is paramount.

We briefly review the functional neuroanatomy of anxiety and discuss the effects of several therapeutic interventions on neural functioning (see Etkin, et al. for further reading) [7].

## Review

The term ‘anxiety disorders’ describes a range of multidimensional phenotypes. Many traits are shared across anxiety disorders. However, key differences exist in underlying cognitive processes that remain to be untangled.

Functional imaging of anxiety disorders

The phenotypic heterogeneity of anxiety disorders is reflected in the heterogeneity of the neuroimaging literature. To take one example, anxiety and depression are highly comorbid [8] and their co-occurrence is known to drive unique brain activation patterns [9]. It is also known that different types of anxiety disorders yield different activation patterns, yet how each of these anxiety disorders subtypes vary when comorbid with depression has not been untangled.

However, we do know that common to three major types of anxiety disorders (post-traumatic stress disorder (PTSD), social anxiety disorder (SAD), and specific phobias (e.g., arachnophobia)) is hyperactivation of the amygdala and insula [10].

The amygdala is one of the most consistently identified regions of hyperactivity in anxiety [11] with its interactional behavior varying across anxiety disorder subtypes [10]. The amygdala serves several major roles including reward learning, unpredictability processing, salience determination in the setting of emotional and social stimuli, and broader stimulus valuation [11-12]. It is theorized that amygdalar dysfunction may drive the inappropriate threat perception and emotional dysregulation believed to lie at the heart of many anxiety disorders.

The clinical manifestation of anxiety is often preceded by what is known as an anxious temperament (AT). Rhesus monkeys are a well-validated primate model of AT and 18-fluorodeoxyglucose-positron emission tomography (FDG-PET) in young rhesus macaques has shown that activity in the lateral division of the central nucleus (CeL) of the dorsal amygdala and in the anterior hippocampus predicts all examined measures of AT [13].

Of interest, the fusiform gyrus appears to hold significant influence over the amygdala in the emotional face-processing of SAD patients. The effect is most profound for viewing fearful faces and, within this condition, activation of the fusiform gyrus was negatively correlated with social anxiety scores and other avoidance-related behavioral assessments [14].

Inappropriately severe and prolonged anticipation of negative events is posited as a common cognitive problem in anxiety disorders. The anticipation of negative outcomes (eg., aversive pictures) recruits a neural network that includes the anterior cingulate cortex (ACC), insula, amygdala, dorsolateral prefrontal cortex (dlPFC), parahippocampal gyrus, and the medial aspects of the bilateral orbitofrontal cortex (OFC) [15-16].

The ACC, together with the insula, are increasingly understood to constitute a “fear network” [17]. Among other functions, ACC is involved in conflict-monitoring and fear learning. Functional magnetic resonance imaging (fMRI) investigation has shown trait-anxiety levels to be inversely correlated with task-related rostral ACC (rACC) activation when viewing affective faces [18]. Trait anxiety has also been correlated positively with ACC activation and negatively with functional connectivity between the ACC and lateral PFC (lPFC) in an emotional conflict task [19].

The insula is thought to play a significant role in the dysfunctional anticipatory processing of anxious individuals [20], which is unsurprising given its role in effective and interoceptive processing [21]. Compared to anxiety-normative (AN) controls, anxiety-prone (AP) individuals demonstrate greater bilateral insular activation during the anticipation of aversive stimuli (pictures of snakes and spiders). This abnormal insular activation is associated with reduced activation of the superior and medial frontal gyrus [20]. Other analyses have specified the right anterior insula (AI) and the left dlPFC to be regions of heightened activity in the anticipation of aversive stimuli in AP patients, with measures of anxiety correlating with greater activation of the amygdala and AI in response to emotional faces [8].

Abnormal anticipatory processing has also been attributed to hyperactivation of the right amygdala and the bed nucleus of the stria terminalis (BNST) [16]. In children with a generalized anxiety disorder (GAD), hyperactivity in the right amygdala during anticipation of aversive images was positively correlated with symptom severity [22].

The PFC is another region of interest in anxiety disorders. Among several roles, the ventrolateral PFC (vlPFC) is known to be activated upon the presentation of emotional distractors during a working memory task [23]. Examination of SAD patients found hypoactivation of the vlPFC in a verbal fluency task, with a negative correlation between vlPFC activation and social avoidance [24].

Researchers at Cambridge found that lesions of the vlPFC in the common marmoset result in increased anxiety characteristics [25]. The same research group found that lesions to either the vlPFC or the anterior OFC result in increased anxiety-related responses to a mock snake [26].

Among the attentional deficits that define anxiety disorders, anxious individuals show an attentional bias (AB) towards threat, with increased vigilance toward threatening stimuli and a decreased ability to disengage from said threats during visual search tasks [27]. Transcranial direct current stimulation (tDCS) of the right dlPFC has been shown to induce attentional impairments similar to those noted in emotional disorders such as anxiety, suggesting a causal role of the dlPFC in anxiety disorders [28]. Meanwhile, anodal tDCS over the left dlPFC significantly decreases the attentional bias for social threat associated with SAD [29].

As previously mentioned, anxiety disorder subtypes display different patterns of brain activation. One such difference lies in PTSD patients. Etkin and colleagues showed PTSD patients demonstrate both regional hypo- and hyperactivity, while SAD and specific phobia patients only showed regional hyperactivity. In comparison to SAD and specific phobia individuals, PTSD patients have significant hypoactivation in the medial PFC (mPFC), rACC and dorsal ACC (dACC), and thalamus. SAD and specific phobia individuals showed more common hyperactivation in the amygdala and insula [10]. However, it was only in PTSD that amygdalar activation was positively associated with symptom severity [30]. mPFC activity [31], and more specifically, ventromedial PFC (vmPFC) activity were also found to positively correlate with symptom severity in PTSD [30].

Obsessive-compulsive disorder (OCD) is characterized by obsessive thought patterns and compulsions (eg., hand-washing, tapping) that becomes debilitating to a patient’s ability to live a normal life. Increased resting OFC and ACC activation has been observed in OCD [32] and anxiety symptoms correlate with ACC hyperactivity [33].

A final distinction of note has been drawn between anxious apprehension (worry) and anxious arousal (somatic anxiety). Engels, et al. describe anxious apprehension as being akin to worry or anticipatory anxiety, with anxious arousal more resembling fear or panic [9]. This distinction has been demonstrated via neuroimaging, with greater right frontal activity associated with anxious apprehension and the contralateral side with anxious arousal [34]. Specifically, anxious apprehension is associated with greater activation in the left IFG and inferior temporal gyrus (ITG), while anxious arousal is associated with less leftward IFG asymmetry. When presented with negative emotion words, individuals with anxious arousal demonstrate greater right-hemisphere temporoparietal activity [35].

Therapeutic interventions for anxiety disorders

Pharmacological therapies

Selective serotonin reuptake inhibitors (SSRI), serotonin-noradrenaline reuptake inhibitors (SNRI), and benzodiazepines are among the most typical pharmacological treatments for anxiety disorders. SSRIs and SNRIs work by inhibiting reuptake pumps on the membrane of presynaptic neurons, increasing the amount of serotonin and norepinephrine in the synaptic cleft available for post-synaptic action.

Citalopram, an SSRI, has been shown to influence neural changes in anxiety disorder patients [36], driving the attenuation of the lateral OFC and right amygdala to aversive faces [37]. Three weeks of escitalopram, another SSRI, decreased activation of the bilateral posterior and middle insula and the mPFC during aversive anticipation [38]. Of interest, pre-treatment activation of the ACC to neutral and aversive stimuli is associated with greater reductions in anxiety after eight weeks of treatment with venlafaxine (SNRI) [39]

Pregabalin affects the brain through a mechanism that ultimately leads to, among other things, the upregulation of GABA, an inhibitory neurotransmitter. Treatment with pregabalin has been shown to attenuate activation of the left amygdala and anterior insula and increases in ACC activation during the anticipation and processing of emotional images [40].

Psychological therapies

Among many therapeutic psychological programs, cognitive behavioral therapy (CBT) has been gaining traction in response to significant outcomes associated with the treatment. In OCD patients, CBT has been shown to induce functional changes in the activation of the putamen, cerebellum and hippocampus [32], subgenual ACC [30], the right head of the caudate nucleus [41], and right dACC [42]. These CBT-driven changes are all correlated with anxiety symptom improvement.

Exposure therapy has traditionally been controversial due to discrepancies in the practitioner’s understanding and utilization of the treatment. That aside, two weeks of exposure to spiders has been shown to reduce hyperactivity in the amygdala, ACC, and insula of phobia-specific anxiety patients [43].

Yet another proposed psychological therapy is mindfulness meditation. Zeidan and colleagues trained participants in mindfulness meditation and then compared the efficacy of the treatment to a control condition in which participants were asked to attend to their breath (ATB). Compared to the ATB group, mindfulness meditation resulted in anxiety relief that correlated with significantly greater activation of the ACC, vmPFC, and AI [44].

Electrical stimulation therapies

One characteristic of anxiety disorders is the AB for threat that is known to contribute to the perseverance of the condition. As anodal tDCS over the left dlPFC has been shown to attenuate the maintenance of the AB for threat; tDCS has presented itself as an interesting therapeutic avenue in anxiety disorders [29, 45]. Several reviews of the literature have found evidence for an anxiolytic effect of repetitive transcranial magnetic stimulation (rTMS) [46-48]. The authors do however note the limitations of many of the studies examining rTMS in the context of anxiety, and further research is required for both these treatments.

Deep brain stimulation (DBS) of the BST has been shown to be safe and effective in treating cases of severe, treatment-resistant OCD [49]. The efficacy of such a treatment has also been proved in a rodent model of non-OCD anxiety, where electrical stimulation of the BST was found to reduce the rodent’s contextual anxiety [50]. However, more work is required before DBS may be used on a large scale for anxiety patients.

## Conclusions

Owing to the heterogeneous and highly comorbid nature of anxiety disorders, a thorough understanding of the neural underpinnings of the disease remains beyond our reach. As with any cognitive process, the complexity of regional and network interactions at a neural level, combined with the limitations of our current methodologies, means such an understanding is not on the immediate horizon. However, significant progress has been made in the past two decades towards developing a more robust picture of anxiety disorders. From this work, promising therapies such as DBS, tDCS, and rTMS have emerged and will continue to be refined in the coming years.
